# FMDNet: An Efficient System for Face Mask Detection Based on Lightweight Model during COVID-19 Pandemic in Public Areas

**DOI:** 10.3390/s23136090

**Published:** 2023-07-02

**Authors:** J. V. Bibal Benifa, Channabasava Chola, Abdullah Y. Muaad, Mohd Ammar Bin Hayat, Md Belal Bin Heyat, Rajat Mehrotra, Faijan Akhtar, Hany S. Hussein, Debora Libertad Ramírez Vargas, Ángel Kuc Castilla, Isabel de la Torre Díez, Salabat Khan

**Affiliations:** 1 Department of Computer Science and Engineering, Indian Institute of Information Technology Kottayam, Kottayam 686635, India; benifa@iiitkottayam.ac.in (J.V.B.B.);; 2 Department of Studies in Computer Science, Mysore University, Manasagangothri, Mysore 570006, India; abdullahmuaad9@gmail.com; 3 M.A.H. Inter College, Deoria 274408, India; ammarhayat97@gmail.com; 4 IoT Research Center, College of Computer Science and Software Engineering, Shenzhen University, Shenzhen 518060, China; 5 Department of Examination and Analysis, Amity University, Noida 201303, India; 6 School of Computer Science and Engineering, University of Electronic Science and Technology of China, Chengdu 610054, China; faijanakhtar98@gmail.com; 7 Electrical Engineering Department, Faculty of Engineering, King Khalid University, Abha 61411, Saudi Arabia; 8 Electrical Engineering Department, Faculty of Engineering, Aswan University, Aswan 81528, Egypt; 9 Higher Polytechnic School, Universidad Europea del Atlántico, Isabel Torres, 39011 Santander, Spain; 10 Department of Engineering and Projects, Universidad Internacional Iberoamericana, Campeche 24560, Mexico; 11 Facultade de Engenharias, Universidade Internacional do Cuanza, Cuito EN250, Angola; 12 School of Engineering, Fundación Universitaria Internacional de Colombia, Bogotá 11001, Colombia; 13 Higher Polytechnic School, Universidad de La Romana, La Romana 22000, Dominican Republic; 14 Department of Signal Theory and Communications, University of Valladolid, 47011 Valladolid, Spain

**Keywords:** artificial intelligence, COVID-19, deep learning, FaceMask, MobileNetV2, pandemic, SARS CoV-2, surveillance, World Health Organization

## Abstract

A new artificial intelligence-based approach is proposed by developing a deep learning (DL) model for identifying the people who violate the face mask protocol in public places. To achieve this goal, a private dataset was created, including different face images with and without masks. The proposed model was trained to detect face masks from real-time surveillance videos. The proposed face mask detection (FMDNet) model achieved a promising detection of 99.0% in terms of accuracy for identifying violations (no face mask) in public places. The model presented a better detection capability compared to other recent DL models such as FSA-Net, MobileNet V2, and ResNet by 24.03%, 5.0%, and 24.10%, respectively. Meanwhile, the model is lightweight and had a confidence score of 99.0% in a resource-constrained environment. The model can perform the detection task in real-time environments at 41.72 frames per second (FPS). Thus, the developed model can be applicable and useful for governments to maintain the rules of the SOP protocol.

## 1. Introduction

The primary and essential precautions against COVID-19 have been maintaining distance between people and wearing a mask [1]. The efficient way to curb the spread of COVID-19 according to many health experts is social distancing [2]. This involves consciously maintaining a certain distance between individuals to prevent the spread of the virus. This measure dramatically minimizes the risk of contracting COVID-19. This practice reduces the rate and extent of transmission in a community by decreasing contact with those who are infected. An experiment was conducted to estimate the efficiency of face masks. It was discovered that each person produces hundreds of droplets (varying in size from 20 to 500 µm), but almost all of the droplets were blocked when the other person was wearing a face mask [3]. In another experiment, wearing a surgical mask was proven to decrease the number of viral particles [4]. A recent study explored the effect of mask wearing on the growth rate of COVID-19 in 15 states of the USA. It was shown that there was a decrease in the daily growth rate of COVID-19 [5]. Medical experts and the World Health Organization (WHO) have continuously recommended wearing face masks to reduce active cases of COVID-19 across the globe [6]. Therefore, each citizen has to follow these guidelines. However, people avoid wearing face masks due to a lack of awareness (such as in underdeveloped countries) and the various discomforts that this causes, such as sweating and the inability to breathe correctly. Therefore, government authorities have faced many challenges in monitoring people to maintain these standard operating procedures (SOP) in public places such as hospitals and other forums.

The global COVID-19 coronavirus outbreak has driven a rise in the popularity of wearing face masks in public. People used masks to protect their health from air pollution prior to COVID-19. Scientists have demonstrated that using a face mask prevents the rapid spread of COVID-19 [3]. COVID-19 is very recent virus affecting human health, which caused a pandemic [7]. The World Health Organization designated COVID-19 as a worldwide epidemic in 2020 due to its quick spread. Reference [8] claimed that, in less than six months, COVID-19 infected over five-million people in 188 different countries. Close contact and congested or overcrowded environments are both conducive to the virus’s propagation. The coronavirus outbreak has spurred remarkable levels of international scientific collaboration.

To predict the dispersion of COVID-19, to have an early warning system for pandemics, and to categorize sensitive people, researchers and physicians can use Machine Learning (ML) to analyze enormous amounts of data. To combat and anticipate new illnesses, the delivery of healthcare requires investment in cutting-edge technologies such as AI, the Internet of Thing (IoT), big data, and ML. The power of AI is being used to combat the COVID-19 pandemic, such as the identification of COVID-19 in medical chest X-rays [9,10], in order to better understand infection rates and trace and swiftly diagnose infections. The spread and transmission of COVID-19 have resulted in a number of concerns and problems for policymakers [11]. In several nations, wearing a face mask in public is required by law. These regulations and legislation were created in response to the exponential rise in incidents and fatalities across several domains. Monitoring huge gatherings of individuals is becoming increasingly challenging, however. Anyone who is not covering their face must be recognized through a surveillance procedure. The Paris Metro system’s security cameras in France have been updated with new AI software capabilities to ensure that users are wearing face masks. The software’s French developer, DatakaLab, anonymized the statistical data, which might assist law enforcement in anticipating COVID-19 outbreaks, rather than identifying or detaining persons who are not wearing masks [12].

ML and DL techniques are extensively used for object detection and recognition problems. In this section, ML and DL models used for Face Mask Detection (FMD) problems are reviewed, and their limitations are summarized. Farrugia et al. [6] built a parallel Convolutional Face Finder (CFF) algorithm for the real-time face detection problem, and this algorithm can process 127 Quarter Video Graphics Array (QVGA) images per second. A reconfigurable model for rotation-invariant multi-view face detection based on a novel two-stage boosting was proposed by Xu et al. [13]. Here, they designed a detector for a tree structure, which established several detector nodes. In addition to this, a boosting algorithm was used for training purposes, such as the robust classifier in every detector node being the combination of multiple two-stage weak classifiers.

The advantage of a fast face detector is that it is suitable for programmable devices and has a homogeneous structure. Zuo et al. [14] presented a face detector using a hierarchical ensemble. Hong et al. [15] introduced both phone-to-face and face detection in real-time. The algorithm retrieves two face regions based on the data estimated from experiments and identifies the face region using vertical and horizontal histograms. Sun et al. [16] designed a DL-based face detection model tested on an FDDB benchmark. The proposed work was more efficient than the traditional faster RCNN model in face detection. Xiao et al. [17] presented a face detection scheme using the positioning of the optimal occlusion area (POOA) algorithm. This algorithm employs the Haar feature, principal component analysis (PCA), and AdaBoost. It provided good results for face detection. Sundararajan and Biswas (2019) [18] developed different face detection methods by enhancing the images. The research was tested on an IDEAL-LIVE Distorted Face Database dataset and provided good results. Zhang et al. [19] proposed a feature agglomeration network (FANet) to build a novel single-stage face detector. The proposed model was tested on several datasets and achieved good results. Guo et al. (2020) [20] proposed a method for face detection based on complete discriminative features associated with a CNN. This approach is useful for face detection and had outstanding results. Sen and Sawant [21] proposed a face mask detection system using the MobileNet V2 model, trained on 9000 face images with and without masks, and the accuracy was 79%. Rahman et al. [22] proposed a deep learning architecture for the FMD task trained on 858 images and obtained an accuracy of about 98.7%.

Previously, Khandelwal et al. [23] developed a DL model that was trained on more than 300 images with masks and 460 images without masks. In [24], the authors proposed a method to identify the condition of face mask wearing by using a classification network with image super-resolution to prevent COVID-19. Jiang et al. [25] proposed a face mask detector. Their model used ResNet and MobileNet on a dataset of 7959 images. Li et al. [26] proposed a method of HGL for the classification of head pose. Their model achieved an accuracy equal to 93.64%. Matthias et al. [27] proposed an FMD model. They used a dataset that was used to detect the main facial features such as the nose, eyes, and mouth. A different number of studies have been proposed for head pose estimation and FMD using several deep learning models such as FSA-Net [28], FaceMaskNet [29], and ResNet [30]. Sethi et al. [31] proposed a deep-learning-based approach for detecting masks over faces in public places. The proposed work handled occlusions utilizing an ensemble for different pre-processing methods in one and two stages. Gupta et al. [32] developed a human face mask detection method from images and videos using the deep learning concept. They used the Region-based Convolutional Neural Network (RCNN) with ResNet-152 as a base model for detecting the masks and action recognition in public places, which can help manage social distancing to mitigate COVID-19. Ullah et al. [33] proposed a novel DeepMaskNet model that detects face masks and performs masked face recognition. Teboulbiat et al. [34] explored a comparative study for face masks when they used different pre-trained models. The evaluation results achieved a confidence score of 100%. Goyal et al. [35] (2021) proposed a face mask detection approach using a Convolutional Neural Network (CNN) based model to handle the task of the presence or absence of a mask on the given input image or video sequence. The approach was able to attain 98% accuracy for the detection part, compared with existing CNN-based architectures, such as the Visual Geometry Group (VGG), MobileNet, DenseNet, and Inception models. Furthermore, the Resnet-based Single-Shot Detector framework model was used to identify faces. In other studies, Mestetskiy et al. [36] proposed biomedical image classification and detection using image processing and computational geometry [37]. The DL-based face mask detection task plays an essential role in various scenarios such as public transport, workplace management, schools, and hospitals. 

In the present research work, a novel deep learning architecture, FMDNet, was designed to mitigate the challenges identified regarding the state-of-the-art methods. The proposed approach, an AI-based “FMDNet” model, was developed to identify people violating the COVID-19 protocols by not wearing masks or wearing them incorrectly. This model first scans the complete face and detects and recognizes the facial landmarks. Thereafter, the region of interest is identified and selected based on the facial landmarks for the detection of the mask. If a mask is not found, it raises an alarm. If the person is wearing a mask, the system scans to detect if the mask is being worn properly or not, and if the mask is found to be worn inappropriately, it raises an alarm. Hence, the “FMDNet” model detects people who do not wear face masks or improperly wear face masks. The contributions of our proposed work are summarized as follows:

- We designed a novel FMDNet model based on deep learning for the detection of face masks.
- We designed and developed a face mask recognition system based on computer vision for real-time deployment.
- Our system achieved the best accuracy compared to existing techniques.
- Our system easily found those people who were not wearing a mask in a gathering place.

## 2. Materials and Methods

**Figure 1:**
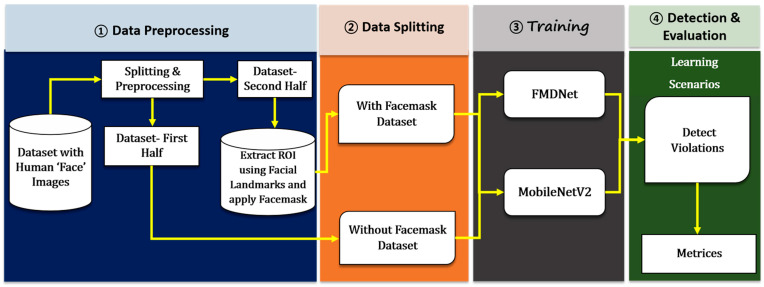
Organizational diagram of the current study.

The high-level workflow of the proposed research work is presented in Figure 1. It consists of mainly three steps: (i) data augmentation, (ii) the development of the proposed FMDNet, and (iii) the model building using FMDNet. 

### 2.1. Datasets

#### 2.1.1. Pins Face Recognition Dataset

This dataset was collected from *Kaggle* (https://www.kaggle.com/hereisburak/pins-face-recog, accessed on 1 October 2022) for facial recognition purposes [38]. A detailed description of the dataset is given in Table 1.

#### 2.1.2. Omkargurav Face-Mask Dataset

The omkargurav face-mask dataset is also available online and as open-access on *Kaggle* (https://www.kaggle.com/datasets/omkargurav/face-mask-dataset, accessed on 1 November 2022). We developed a face-mask-detection model that was trained on 7553 RGB with-mask and without-mask images. There were 3725 images of faces with a mask and 3828 images of faces without a mask. The training accuracy on the custom CNN architecture model was 94%, while the validation accuracy was 96%. The dataset is available online.

#### 2.1.3. Labelled Faces in the Wild Dataset

The Labelled Faces in the Wild (LFW) dataset (https://www.kaggle.com/datasets/muhammeddalkran/lfw-simulated-masked-face-dataset, accessed on 1 December 2022) was used to create our dataset. Images of notable individuals were taken from the website and are part of the LFW dataset. The collection includes 5713 unique faces, totaling 13,117. The training dataset includes 5713 images of people wearing masks out of 13,027 faces. The testing dataset comprises 70 masked faces of 48 people. The dataset is available online.

#### 2.1.4. Real-World-Masked-Face-Dataset

People around the nation wear masks as a result of the COVID-19 pandemic, which has spread across the globe, and several samples of masked faces can be found (https://github.com/X-zhangyang/Real-World-Masked-Face-Dataset, accessed on 20 March 2023). In order to gather data resources for potential future intelligent administration and control of comparable public safety incidents, they, as a result, have generated the largest masked-face dataset in the world. When the community was under lockdowns, appropriate masked face detection and identification algorithms were created based on a masked face dataset to assist those entering and leaving the community. They created and trained a face–eye-based multi-granularity masked face recognition model using the datasets they created. The dataset’s face identification accuracy is over 95%. The dataset is available online.

#### 2.1.5. YOLO-Medical-Mask-Dataset

This dataset is based on Mikolaj Witkowski’s dataset (https://www.kaggle.com/datasets/gooogr/yolo-medical-mask-dataset, accessed on 2 April 2023). All these images have been translated into jpg format. At the same time, the images are only of people properly wearing medical masks. Mikolaj Witkowski’s dataset gives more details about these data. The dataset is available online.

### 2.2. Data Augmentation

**Figure 2:**
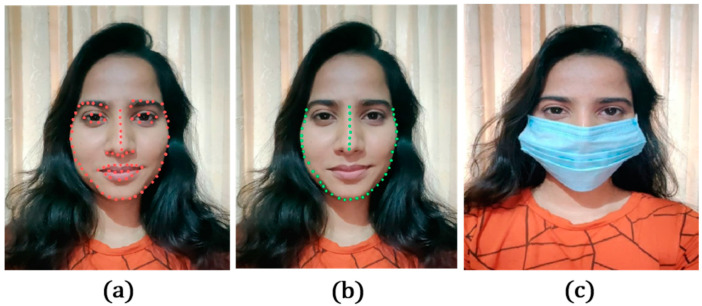
An illustration of the images indicates (**a**) facial landmark detection, (**b**) regions of interest, and (**c**) after applying a face mask, in the proposed study.

Subsequently, the faces in the images were detected by applying facial landmarks [39] and the regions of interest, i.e., the landmarks of the detected “nose” and “chin”, to apply a transparent face mask over the face. The illustration of the work is shown in Figure 2. To place the mask over the face, the four main points were detected from the facial landmarks, namely: (i) the nose’s top, i.e., the topmost point on the bridge of the nose detected in the previous step, (ii) the chin’s bottom, i.e., the bottommost points of the chin detected in the previous step, (iii) the left of the chin, i.e., the leftmost point of the chin detected in the previous step, and (iv) the right of the chin, i.e., the rightmost point of the chin detected in the previous step. After obtaining these four points, resizing was performed by calculating the new length and width for the transparent mask image as in Equations (1) and (2) below:

(1) length_new=nose_top−chin_bottom

(2) width_new=chin_right−chin_left.

Then, the center coordinates were computed for positioning the mask as in Equations (3) and (4) below:

(3) x=(nose_top_x+chin_botton_x)/2

(4) y=(nose_top_y+chin_botton_y)/2

### 2.3. Proposed Model

Deep learning models require considerable resources to work efficiently. Still, the majority of the problems in the natural world require lightweight applications so that they can be deployed in a resource-constrained environment. The MobileNet deep learning model is highly lightweight, so it operates efficiently in a resource-constrained environment. The MobileNets vary from traditional CNNs through the practice of depthwise separable convolution. The proposed model was based on the enhanced MobileNetV2. They have been used to expand the input representations [30]. In addition, non-linearities must be removed to maintain representational power. The proposed FMDNet model has some information such as the structural details, dimensions, and architecture, as shown in Table 2 and Table 3, respectively. The FMDNet architecture includes two types: (i) a residual block, which has a stride of one, and (ii) a block for downsizing with a stride of two. Each block of these blocks has three different layers. The operation of the 1 × 1 convolution is performed in the first layer along with Rectified Linear Unit (ReLU). The operation of depth wise convolution is performed in the second layer. The third layer is similar to the first layer (operation of 1 × 1 convolution), barring non-linearity. The speculation is that if ReLU is used again, the deep networks only have the power of a linear classifier on the non-zero volume part of the output domain. Additionally, a head of five fully connected layers, including one average pooling layer with a pool size of 5, a flattening layer, and 3 dense layers, were added on top of the model.

The architectural highlights are summarized as follows:

- A kernel size of 3 × 3 was used for the spatial convolutions.
- The total number of parameters was 2,619,074, out of which 2,584,962 were trainable parameters and 34,112 were non-trainable parameters.
- The network was trained on an NVIDIA-SMI 455.32.00, with 32 as the batch size.

## 3. Results and Discussion

### 3.1. Experimental Environment

The architecture was trained on the CoLab environment with 12GB of RAM and GPU settings. The experiments were carried out using many libraries, such as OpenCV–4.5.5, Keras 2.12.0, and TensorFlow v2.12.0. MobileNetV2 and FMDNet were evaluated using the accuracy, recall, precision, F1-score, and loss performance metrics in a real-time environment. The customized Dataset-1 and the benchmark datasets, Dataset-2, Dataset-3, Dataset-4, and Dataset-5, were split into two portions: 80% and 20%, respectively. MobileNetV2 and FMDNet were integrated with YOLOV5 for real-time testing and deployment. The first portion was used for training, and the second portion was used for testing. The training process was performed for 100 epochs, and the batch size was kept at 32.

### 3.2. Confidence and Frames Per Second

During the real-time implementation of the proposed model, after providing the input to the face detector model, firstly, it returns the coordinates of the bounding box of the detected face from the input image along with the confidence score of the detection. The confidence score represents how likely the box is to contain an object of interest and how accurate the bounding box is. If no object exists in that cell, the confidence score should be zero. A confidence score was used as a threshold with a value of 0.5 as the default to filter the unwanted data. The bounding box coordinates of the detected face were set to be the input for the classifier. For each input, the classifier returns the prediction score for both the mask and no-mask classes. The higher score was considered to be the final output. The frames per second (FPS) represent how fast the object detection model processed the input images and generated the desired outcome.

In Table 4, the results showed that the detector model had high confidence in detecting faces, scoring 99.99 for no mask and 98.59 for the faces with a mask using the customized Dataset-1. At the same time, the detector model was capable of detecting faces with a mask or no mask with an average of 41.72689 FPS for no mask and 41.9384 FPS for a face with a mask. It was also observed that the FPS obtained were sufficient for real-time feedback. While operating with FMDNet in a resource-constrained environment (without GPU support), a lightweight model of a size of 14 MB can perform well by providing a 20 FPS detection rate with a 97.97 confidence score to be deployed in a real-time environment. In addition to this, FMDNet was evaluated with the public Dataset-2, Dataset-3, Dataset-4, and Dataset-5, and the results were interesting. The accuracy was almost 99.99% and 98.9% with the no-mask and mask categories, respectively. The FPS scores were also found to be optimal for the real-time usage of the model.

### 3.3. Comparison between MobileNetV2 and FMDNet Models

**Figure 3:**
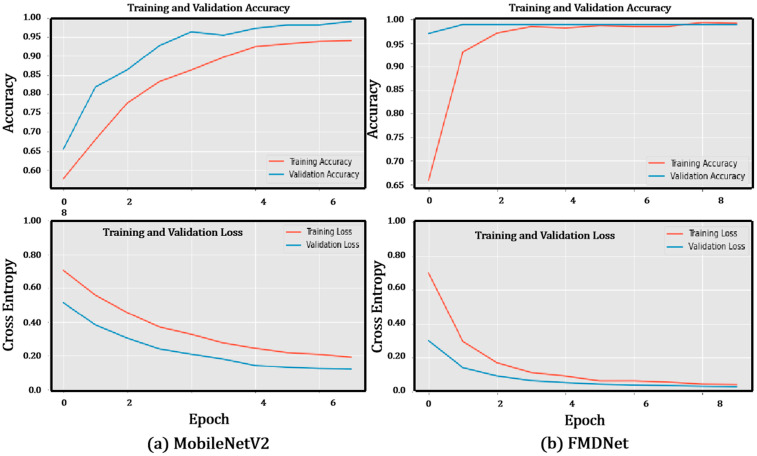
Training and validation related to the accuracy and loss of the (**a**) MobileNetV2 and (**b**) FMDNet models.

The performance of MobileNetV2 vs. FMDNet on the training set and testing set concerning the accuracy and loss are highlighted in this section. Figure 3 highlights the learning curve for MobileNetV2 and FMDNet, respectively, for model learning performance over the epochs. In each epoch, the values of the accuracy and loss metrics represent the learning curves of the model for the training and validation data. The accuracy was also used as an evaluation metric for testing the model. While the curves of both graphs can be considered a good fit, FMDNet provided a better model since the loss and accuracy for both the validation and the training set converged at comparatively better values in contrast to the base model MobileNetV2. In the case of loss, the FMDNet model converged at a lower value than MobileNetV2 for both the validation set and the training set. In the case of accuracy, the FMDNet model converged at a higher value than the base model for both the validation set and the training set. 

**Figure 4:**
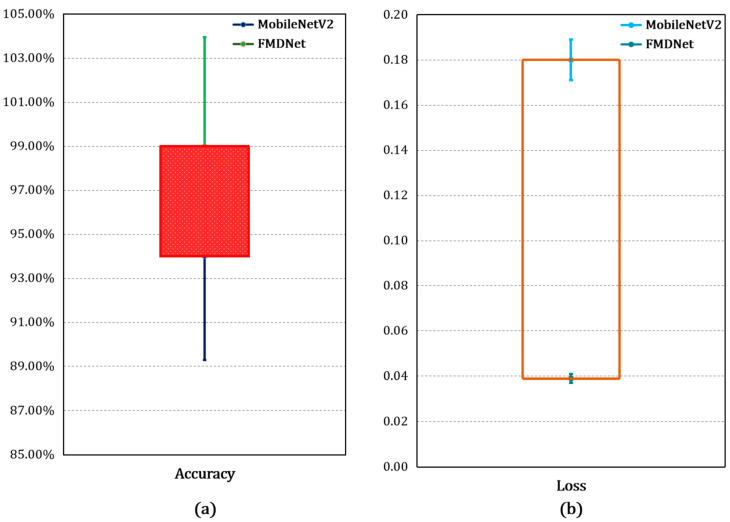
Accuracy and loss on the training dataset using the (**a**) MobileNetV2 and (**b**) FMDNet models.

The accuracy and loss of the training dataset for MobileNetV2 and FMDNet are presented in Figure 4a. This represents several correct decisions made by the FMDNet classifier over the training set. For the same training set, FMDNet (99%) provided a considerable amount of better accuracy than the base model, MobileNetV2, which was only 94%. Figure 4b is the representation of the loss, which in simple terms is the divergence of the predicted probability with the actual result. It was inferred that, for the same training set, FMDNet (0.039) provided a considerable amount of better loss than the base model, MobileNetV2 (0.18). In FMDNet, the layers were designed in such a way to have less floating point operations as compared to the other methods. Here, the number of trainable parameters was also less. With fewer trainable parameters, the model yielded better performance as compared to the state-of-the-art methods. The system worked efficiently and detected faces either with a mask or no mask.

**Figure 5:**
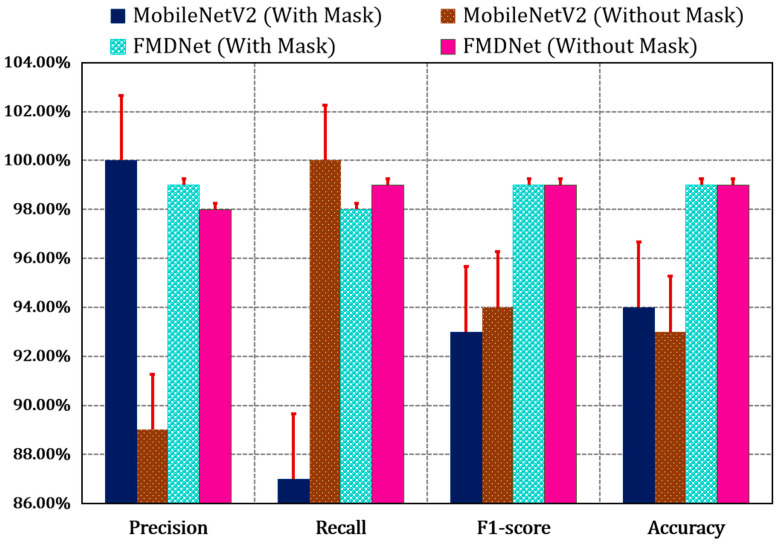
Comparative analysis based on the performance using the MobileNetV2 and FMDNet models.

Figure 5 and Table 5 give the proportion of correct decisions made by FMDNet over the total number of decisions made, and these were calculated over the test set. We can see that, for the same test set, FMDNet (99%) improved the accuracy more than that of the base model, MobileNetV2 (94%). The report consists of some of the significant evaluation metrics such as the precision, recall, F1-score, and accuracy [40,41,42,43,44,45,46,47,48,49] mentioned in Equations (5)–(8). These metrics are based on the true positives, true negatives, false positives, and false negatives.

(5) $$\mathrm{Precision}=\frac{\mathrm{True}\;\mathrm{Positives}}{\mathrm{True}\;\mathrm{Positives}+\mathrm{False}\;\mathrm{Positives}}$$

(6) $$\mathrm{Recall}=\frac{\mathrm{True}\;\mathrm{Positives}}{\mathrm{True}\;\mathrm{Positives}+\mathrm{False}\;\mathrm{Negatives}}$$

(7) $$F1\text{-}\mathrm{score}=\frac{2\times \mathrm{True}\;\mathrm{Positives}}{2\times \mathrm{True}\;\mathrm{Positives}+\mathrm{False}\;\mathrm{Positives}+\mathrm{False}\;\mathrm{Negatives}}$$

(8) $$\mathrm{Accuracy}=\frac{\mathrm{True}\;\mathrm{Positives}+\mathrm{True}\;\mathrm{Negatives}}{\left(\mathrm{True}\;\mathrm{Positives}+\mathrm{True}\;\mathrm{Negatives}\right)+(\mathrm{False}\;\mathrm{Positives}+\mathrm{False}\;\mathrm{Negatives})}$$

### 3.4. Comparison of Proposed and Existing Models

Machine and deep learning approaches are widely utilized for object identification and recognition issues [50,51,52]. For the real-time face detection problem, some researchers have developed the parallel convolutional face finder method, which can analyze 127 QVGA pictures per second. Some have suggested a reconfigurable model for rotation-invariant multi-view face identification based on a unique two-stage boosting, where they created a detector for a tree structure, which creates many detector nodes for training by combining several two-stage weak classifiers. In the presented work, creating a deep learning model was suggested as a new AI-based strategy for finding those who disregard the rules in public settings. To do this, a private dataset was made from many facial photos, both with and without masks. The suggested model was trained to recognize face masks in live surveillance footage. The deep learning classifier was integrated with YOLOv5 for real-time processing. The suggested model can identify infractions (no face mask) in public areas with a promising detection rate of 99.0%. In comparison to other contemporary DL models such as Fine-Grained Structure Aggregation Network (FSA-Net), MobileNetV2, and ResNet, the model exhibited a detection capability of 24.03%, 5.0%, and 24.10%, respectively. The model is resource-effective, has a low mass, and has a confidence score of 99.0%. The prototype can complete the detection job with a frames-per-second (FPS) rate of 41.72 in real-time situations. As a result, governments may be able to adopt and use the established model to uphold the Standard Operating Procedure (SOP) requirements. Table 6 shown below aims to present a comparative analysis of various State-of-The-Art (SOTA) models/techniques used for face mask identification.

### 3.5. Limitations and Future Recommendations

This method may be adjusted to better fit the matching field of vision since it is extremely sensitive to the spatial placement of the camera. These types can be used in conjunction with security cameras in busy public places such as train stations, metros, and office buildings for monitoring compliance with rules and mask-wearing identification. By training on bigger datasets, the learned weights offered by the authors can be further enhanced and used in real-world applications. To make the task of guards easier, we can later add body temperature detection to this system. Additionally, it is hoped that this device will be put in high-population areas that require face mask detectors. This approach may be utilized in any setting where accuracy and precision are crucial to the task at hand, including public spaces, stations, business settings, roadways, malls, and testing facilities. This method may be used for smart city innovation and would hasten the pace of development in many underdeveloped nations. Our examination of the existing situation offers the opportunity to assess the consequences of significant social change or to become better prepared for the next disaster.

## 4. Conclusions

Identifying face masks in streaming videos and photos was the purpose of this work. This article suggested a DL-based model called FMDNet. The method can be used to detect those who disobey the rules of wearing face masks in public. To process live video streams and photos, FMDNet was put into use. The suggested technique was used to extract faces from photos and videos, and it successfully recognized them. In comparison to the state-of-the-art models, FMDNet was superior in terms of deployment in a low-resource environment, the confidence score, the accuracy, and the FPS for real-time feedback. FMDNet can accurately identify mask wearers and non-mask wearers from an input image or frame in a variety of different lighting scenarios and clear environment situations. The average accuracy attained after training the FMDNet model was 99%. By accurately determining whether someone is wearing a mask or not, COVID-19 may be regulated thanks to FMDNet’s straightforward framework and speed. Videos taken in public spaces can also be utilized for mass screenings of people wearing masks or not, making it useful in densely populated public areas. The public authorities can efficiently identify infractions and stop the spread of COVID-19 by using the study that has been presented. This model was tested with a real-time lightweight device and can be deployed for real-time applications. This model can be utilized for biometric purposes and for continuous monitoring systems in schools, colleges, and various scenarios. Furthermore, it can be deployed in public places to monitor crowd clusters.

## Figures and Tables

**Table 1:** Data distribution per class for the pins face recognition dataset.

No.	Class Type	No. of Images	Train	Test
1	With mask	600	300	300
2	Without mask	600	300	300
Total		1200	600	600

**Table 2:** Structural details of the proposed FMDNet model.

Input	Operator	Expansion Factor	No. of Output Channels	Repeating No.	Stride Size
224 × 224 × 3	Conv2d	-	32	1	2
112 ×112 × 32	Bn	1	16	1	1
1122 × 16	Bn	6	24	2	2
562 × 24	Bn	6	32	3	2
282 × 32	Bn	6	64	4	2
142 × 64	Bn	6	96	3	1
142 × 96	Bn	6	160	3	2
72 × 160	Bn	6	320	1	2
72 × 320	Conv2d 1 × 1	-	1280	1	1
72 × 1280	Avgpool 7 × 7	-	-	1	1
1 × 1 × 1280	Conv2d 1 × 1	-	K	-	-

**Table 3:** Dimensions of the proposed FMDNet model.

Layer	Input Dimensions	Output Channels
Conv2d	224 × 224 × 3	32
Bn	112 × 112 × 32	16
Bn	112 × 112 × 16	24
Bn	56 × 56 × 24	32
Bn	28 × 28 × 32	64
Bn	14 × 14 × 64	96
Bn	14 × 14 × 96	160
Bn	7 × 7 × 160	320
Conv2d	7 × 7 × 320	1280
Avgpool	7 × 7 × 1280	1280
Flatten	1 × 1 × 1280	-
Dense 1	1280	256
Dense 2	256	128
Dense 3	128	2

**Table 4:** The confidence score for the proposed study.

Dataset	Dataset No.	Mask/No Mask	Confidence	FPS
Pins Face Recognition Dataset	Dataset-1	No Mask	99.99	41.7268
		Mask	98.59	41.9384
Omkargurav Face-Mask Dataset	Dataset-2	No Mask	99.98	41.8548
		Mask	98.91	41.6729
Labelled Faces in the Wild (LFW) Dataset	Dataset-3	No Mask	99.95	41.9521
		Mask	98.96	41.7603
Real-World-Masked-Face-Dataset	Dataset-4	No Mask	99.92	41.8171
		Mask	98.94	41.9513
YOLO-Medical-mask-Dataset	Dataset-5	No Mask	99.99	41.8607
		Mask	98.91	41.6713

**Table 5:** Evaluation metrics of the FMDNet and MobileNetV2 models for Dataset-1.

No.	Model	Information	Precision	Recall	F1-Score	Accuracy
1	MobileNetV2	With mask	1.00	0.87	0.93	0.94
		Without mask	0.89	1.00	0.94	0.93
2	FMDNet	**With mask**	**0.99**	**0.98**	**0.99**	**0.99**
		**Without mask**	**0.98**	**0.99**	**0.99**	**0.99**

**Table 6:** Comparative analysis of various SOTA models/techniques used for face mask identification FMD Task.

Author	Year	Model	Performance
[21]	2020	Deep learning framework	Accuracy: 79.24%
[22]	2020	Deep learning framework	Accuracy: 98.70%
[23]	2020	Deep learning and classic projective geometry techniques	AUC: 97.6%, precision: 97.00%, recall: 97.00%
[24]	2020	Deep-Learning-based SRCNet	Accuracy: 98.70%
[26]	2020	HGL to deal with the head pose classification with CNN	Front accuracy: 93.64%, side accuracy: 87.17%
[29]	2020	Deep learning method called FaceMaskNet	Accuracy: 98.6%
[30]	2020	Generative adversarial networks (GANs) and support vector machines (SVMs) classifier	Mask sub-challenge: 74.6%
[31]	2021	ResNet50, AlexNet, and MobileNet	Accuracy: 98.2%
[32]	2022	Expanded mask R-CNN	mAP: 80.25
[34]	2022	Deep MaskNet framework-MDMFR	Accuracy: 93.33
[35]	2022	CNN architecture	Accuracy: 98%
**Proposed Model**		FMDNet	Accuracy: **99%**

## Data Availability

We used open-access datasets.

## References

[1] Fauci A.S., Lane H.C., Redfield R.R. (2020). COVID-19—Navigating the Uncharted. N. Engl. J. Med..

[2] Kuitunen I., Artama M., Mäkelä L., Backman K., Heiskanen-Kosma T., Renko M. (2020). Effect of Social Distancing Due to the COVID-19 Pandemic on the Incidence of Viral Respiratory Tract Infections in Children in Finland during Early 2020. Pediatr. Infect. Dis. J..

[3] Feng S., Shen C., Xia N., Song W., Fan M., Cowling B.J. (2020). Rational Use of Face Masks in the COVID-19 Pandemic. Lancet Respir. Med..

[4] Leung N.H.L., Chu D.K.W., Shiu E.Y.C., Chan K.H., McDevitt J.J., Hau B.J.P., Yen H.L., Li Y., Ip D.K.M., Peiris J.S.M. (2020). Respiratory Virus Shedding in Exhaled Breath and Efficacy of Face Masks. Nat. Med..

[5] Li T., Liu Y., Li M., Qian X., Dai S.Y. (2020). Mask or No Mask for COVID-19: A Public Health and Market Study. PLoS ONE.

[6] Mamalet F., Farrugia N., Roux S., Yang F., Paindavoine M. (2008). Design of a Real-Time Face Detection Parallel Architecture Using High-Level Synthesis. EURASIP J. Embed. Syst..

[7] Liu X., Zhang S. (2020). COVID-19: Face Masks and Human-to-Human Transmission. Influenza Other Respi. Viruses.

[8] World Health Organization (WHO) (2020). WHO Coronavirus Disease (COVID-19) Dashboard|WHO Coronavirus Disease (COVID-19) Dashboard.

[9] Loey M., Smarandache F., Khalifa N.E.M. (2020). Within the Lack of Chest COVID-19 X-ray Dataset: A Novel Detection Model Based on GAN and Deep Transfer Learning. Symmetry.

[10] Ting D.S.W., Carin L., Dzau V., Wong T.Y. (2020). Digital Technology and COVID-19. Nat. Med..

[11] Altmann D.M., Douek D.C., Boyton R.J. (2020). What Policy Makers Need to Know about COVID-19 Protective Immunity. Lancet.

[12] Fouquet H. Paris Tests Face-Mask Recognition Software on Metro Riders. https://www.bloomberg.com/news/articles/2020-05-07/paris-tests-face-mask-recognition-software-on-metro-riders%0Ahttps://www.bloomberg.com/news/articles/2020-05-07/paris-tests-face-mask-recognition-software-on-metro-riders?sref=C3P1bRLC.

[13] Xu J., Dou Y., Pang Z. (2009). A Reconfigurable Architecture for Rotation Invariant Multi-View Face Detection Based on a Novel Two-Stage Boosting Method. EURASIP J. Adv. Signal Process..

[14] Zuo F., de With P.H.N. (2007). Cascaded Face Detection Using Neural Network Ensembles. EURASIP J. Adv. Signal Process..

[15] Hong S.H., Lee J.W., Lama R.K., Kwon G.R. (2016). Real-Time Face Detection and Phone-to-Face Distance Measuring for Speech Recognition for Multi-Modal Interface in Mobile Device. Multimed. Tools Appl..

[16] Sun X., Wu P., Hoi S.C.H. (2018). Face Detection Using Deep Learning: An Improved Faster RCNN Approach. Neurocomputing.

[17] Xiao Y., Cao D., Gao L. (2020). Face Detection Based on Occlusion Area Detection and Recovery. Multimed. Tools Appl..

[18] Soundararajan R., Biswas S. (2019). Machine Vision Quality Assessment for Robust Face Detection. Signal Process. Image Commun..

[19] Zhang J., Wu X., Hoi S.C.H., Zhu J. (2020). Feature Agglomeration Networks for Single Stage Face Detection. Neurocomputing.

[20] Guo G., Wang H., Yan Y., Zheng J., Li B. (2020). A Fast Face Detection Method via Convolutional Neural Network. Neurocomputing.

[21] Sen S., Sawant K. (2021). Face Mask Detection for Covid_19 Pandemic Using Pytorch in Deep Learning. IOP Conf. Ser. Mater. Sci. Eng..

[22] Rahman M.M., Manik M.M.H., Islam M.M., Mahmud S., Kim J.H. (2020). An Automated System to Limit COVID-19 Using Facial Mask Detection in Smart City Network. Proceedings of the IEMTRONICS 2020—International IOT, Electronics and Mechatronics Conference.

[23] Khandelwal P., Khandelwal A., Agarwal S., Thomas D., Xavier N., Raghuraman A. (2020). Using Computer Vision to Enhance Safety of Workforce in Manufacturing in a Post COVID World. arXiv.

[24] Qin B., Li D. (2020). Identifying Facemask-Wearing Condition Using Image Super-Resolution with Classification Network to Prevent COVID-19. Sensors.

[25] Fan X., Jiang M. RetinaFaceMask: A Single Stage Face Mask Detector for Assisting Control of the COVID-19 Pandemic. Proceedings of the 2021 IEEE International Conference on Systems, Man, and Cybernetics (SMC).

[26] Li S., Ning X., Yu L., Zhang L., Dong X., Shi Y., He W. (2020). Multi-Angle Head Pose Classification When Wearing the Mask for Face Recognition under the COVID-19 Coronavirus Epidemic. Proceedings of the 2020 International Conference on High Performance Big Data and Intelligent Systems, HPBD and IS 2020.

[27] Matthias D., Managwu C., Olumide O. (2021). Face Mask Detection Application and Dataset. J. Comput. Sci. Its Appl..

[28] Yang T.Y., Chen Y.T., Lin Y.Y., Chuang Y.Y. Fsa-Net: Learning Fine-Grained Structure Aggregation for Head Pose Estimation from a Single Image. Proceedings of the IEEE Computer Society Conference on Computer Vision and Pattern Recognition.

[29] Inamdar M., Mehendale N. (2020). Real-Time Face Mask Identification Using Facemasknet Deep Learning Network. SSRN Electron. J..

[30] Ristea N.C., Ionescu R.T. Are You Wearing a Mask? Improving Mask Detection from Speech Using Augmentation by Cycle-Consistent GANs. Proceedings of the Annual Conference of the International Speech Communication Association, INTERSPEECH 2020.

[31] Sethi S., Kathuria M., Kaushik T. (2021). Face Mask Detection Using Deep Learning: An Approach to Reduce Risk of Coronavirus Spread. J. Biomed. Inform..

[32] Gupta P., Sharma V., Varma S. (2022). A Novel Algorithm for Mask Detection and Recognizing Actions of Human. Expert Syst. Appl..

[33] Ullah N., Javed A., Ali Ghazanfar M., Alsufyani A., Bourouis S. (2022). A Novel DeepMaskNet Model for Face Mask Detection and Masked Facial Recognition. J. King Saud Univ.-Comput. Inf. Sci..

[34] Teboulbi S., Messaoud S., Hajjaji M.A., Mtibaa A. (2022). Real-Time Implementation of AI-Based Face Mask Detection and Social Distancing Measuring System for COVID-19 Prevention. Sci. Program..

[35] Goyal H., Sidana K., Singh C., Jain A., Jindal S. (2022). A Real Time Face Mask Detection System Using Convolutional Neural Network. Multimed. Tools Appl..

[36] Mestetskiy L.M., Guru D.S., Benifa J.V.B., Nagendraswamy H.S., Chola C. (2022). Gender Identification of Drosophila Melanogaster Based on Morphological Analysis of Microscopic Images. Vis. Comput..

[37] Chola C., Benifa J.V.B., Guru D.S., Muaad A.Y., Hanumanthappa J., Al-Antari M.A., AlSalman H., Gumaei A.H. (2022). Gender Identification and Classification of Drosophila Melanogaster Flies Using Machine Learning Techniques. Comput. Math. Methods Med..

[38] Burak Pins Face Recognition. https://www.kaggle.com/datasets/hereisburak/pins-face-recognition/metadata%0Ahttps://www.kaggle.com/hereisburak/pins-face-recog.

[39] Kazemi V., Sullivan J. One Millisecond Face Alignment with an Ensemble of Regression Trees. Proceedings of the IEEE Computer Society Conference on Computer Vision and Pattern Recognition 2014.

[40] Pal R., Adhikari D., Bin Heyat B., Ullah I., You Z. (2023). Yoga Meets Intelligent Internet of Things: Recent Challenges and Future Directions. Bioengineering.

[41] Bin Heyat B., Akhtar F., Abbas S.J., Al-Sarem M., Alqarafi A., Stalin A., Abbasi R., Muaad A.Y., Lai D., Wu K. (2022). Wearable Flexible Electronics Based Cardiac Electrode for Researcher Mental Stress Detection System Using Machine Learning Models on Single Lead Electrocardiogram Signal. Biosensors.

[42] Bin Heyat B., Akhtar F., Khan A., Noor A., Benjdira B., Qamar Y., Abbas S.J., Lai D. (2020). A Novel Hybrid Machine Learning Classification for the Detection of Bruxism Patients Using Physiological Signals. Appl. Sci..

[43] Qadri S.F., Lin H., Shen L., Ahmad M., Qadri S., Khan S., Khan M., Zareen S.S., Akbar M.A., Bin Heyat M.B. (2023). CT-Based Automatic Spine Segmentation Using Patch-Based Deep Learning. Int. J. Intell. Syst..

[44] Alphonse A.S., Benifa J.V.B., Muaad A.Y., Chola C., Bin Heyat B., Murshed B.A.H., Samee N.A., Alabdulhafith M., Al-Antari M.A. (2023). A Hybrid Stacked Restricted Boltzmann Machine with Sobel Directional Patterns for Melanoma Prediction in Colored Skin Images. Diagnostics.

[45] Lai D., Bin Heyat B., Khan F.I., Zhang Y. (2019). Prognosis of Sleep Bruxism Using Power Spectral Density Approach Applied on EEG Signal of Both EMG1-EMG2 and ECG1-ECG2 Channels. IEEE Access.

[46] Bin Heyat B., Lai D., Khan F.I., Zhang Y. (2019). Sleep Bruxism Detection Using Decision Tree Method by the Combination of C4-P4 and C4-A1 Channels of Scalp EEG. IEEE Access.

[47] Bin Heyat B., Lai D., Wu K., Akhtar F., Sultana A., Tumrani S., Teelhawod B.N., Abbasi R., Kamal M.A., Muaad A.Y. (2022). Role of Oxidative Stress and Inflammation in Insomnia Sleep Disorder and Cardiovascular Diseases: Herbal Antioxidants and Anti-Inflammatory Coupled with Insomnia Detection Using Machine Learning. Curr. Pharm. Des..

[48] Sultana A., Rahman K., Bin Heyat B., Sumbul U., Akhtar F., Muaad A.Y. (2022). Role of Inflammation, Oxidative Stress, and Mitochondrial Changes in Premenstrual Psychosomatic Behavioral Symptoms with Anti-Inflammatory, Antioxidant Herbs, and Nutritional Supplements. Oxid. Med. Cell. Longev..

[49] Qayyum S., Sultana A., Bin Heyat B., Rahman K., Akhtar F., Haq A.U., Alkhamis B.A., Alqahtani M.A., Gahtani R.M. (2023). Therapeutic Efficacy of a Formulation Prepared with *Linum usitatissimum* L., *Plantago ovata* Forssk., and Honey on Uncomplicated Pelvic Inflammatory Disease Analyzed with Machine Learning Techniques. Pharmaceutics.

[50] Teelhawod B.N., Akhtar F., Heyat M.B.B., Tripathi P., Mehrotra R., Asfaw A.B., Al Shorman O., Masadeh M. (2021). Machine Learning in E-Health: A Comprehensive Survey of Anxiety. Proceedings of the 2021 International Conference on Data Analytics for Business and Industry, ICDABI 2021.

[51] Akhtar F., Bin Heyat M.B., Li J.P., Patel P.K., Rishipal, Guragai B. (2020). Role of Machine Learning in Human Stress: A Review. Proceedings of the 2020 17th International Computer Conference on Wavelet Active Media Technology and Information Processing, ICCWAMTIP 2020.

[52] Tripathi P., Ansari M.A., Gandhi T.K., Mehrotra R., Bin Heyat B., Akhtar F., Ukwuoma C.C., Muaad A.Y., Kadah Y.M., Al-Antari M.A. (2022). Ensemble Computational Intelligent for Insomnia Sleep Stage Detection via the Sleep ECG Signal. IEEE Access.

